# Sex differences in impact of cumulative systolic blood pressure from childhood to adulthood on albuminuria in midlife: a 30-year prospective cohort study

**DOI:** 10.1186/s12889-023-15613-y

**Published:** 2023-04-11

**Authors:** Dan Wang, Pu-qing Kou, Yue-yuan Liao, Ke-ke Wang, Yu Yan, Chen Chen, Chao Chu, Yang Wang, Ze-Jiaxin Niu, Qiong Ma, Yue Sun, Jian-jun Mu

**Affiliations:** 1 Department of Cardiology, First Affiliated Hospital of Medical School, Xi’an Jiaotong University, No. 277, Yanta West Road, Xi’an, 710061 China; 2 Key Laboratory of Molecular Cardiology of Shaanxi Province, Xi’an, China; 3 Key Laboratory of Environment and Genes Related to Diseases (Xi’an Jiaotong University), Ministry of Education, Xi’an, Shaanxi 710061 China

**Keywords:** Albuminuria, Cumulative blood pressure, Middle-aged, Prospective studies, Sex characteristics

## Abstract

**Background and objectives:**

Albuminuria is recognized as being a predictor of cardiovascular and renal disease. We aimed to identify the impact of the long-term burden and trends of systolic blood pressure on albuminuria in midlife, as well as to explore sex differences concerning this relationship.

**Methods:**

This longitudinal study consisted of 1,683 adults who had been examined 4 or more times for blood pressure starting in childhood, with a follow-up time period of 30 years. The cumulative effect and longitudinal trend of blood pressure were identified by using the area under the curve (AUC) of individual systolic blood pressure measurement with a growth curve random effects model.

**Results:**

Over 30 years of follow-up, 190 people developed albuminuria, including 53.2% males and 46.8% females (aged 43.39 ± 3.13 years in the latest follow-up). The urine albumin-to-creatinine ratio (uACR) values increased as the total and incremental AUC values increased. Additionally, women had a higher albuminuria incidence in the higher SBP AUC groups than men do (13.3% for men vs. 33.7% for women). Logistic regression showed that the ORs of albuminuria for males and females in the high total AUC group were 1.34 (0.70–2.60) and 2.94 (1.50–5.74), respectively. Similar associations were found in the incremental AUC groups.

**Conclusions:**

Higher cumulative SBP was correlated with higher uACR levels and a risk of albuminuria in middle age, especially in women. The identification and control of cumulative SBP levels from an early age may assist in reducing the incidences of renal and cardiovascular disease for individuals in later life.

**Supplementary Information:**

The online version contains supplementary material available at 10.1186/s12889-023-15613-y.

## Introduction

Albuminuria is an early biomarker of endothelial dysfunction and albumin excretion, has been confirmed to be a powerful predictor of cardiovascular and renal disease risks in diabetic and nondiabetic patients [1–3]. Albuminuria has a high prevalence, with a prevalence ranging from 10 to 40% in people with hypertension and diabetes; additionally, it may precede the onset of traditional CVD risk factors [4, 5]. Extensive evidence has demonstrated that albuminuria is associated with higher cardiovascular and all-cause mortality, heart failure, end-stage renal disease and progression of kidney disease [6–8]. Therefore, it is important to identify modifiable risk factors for albuminuria, especially in early life, to improve albuminuria in midlife.

Hypertension is one of the leading risk factors in cardiovascular and renal outcomes, [9, 10] and is directly related to the progression of heart failure and chronic kidney disease (CKD) [11]. Currently, the incidence and prevalence of isolated systolic hypertension are increasing among young and middle-aged people [12]. Cross-sectional studies have confirmed that blood pressure is associated with albuminuria and increased microalbuminuria excretion in adults, which is not solely mediated through vascular damage [13, 14]. Prospective longitudinal cohorts support the viewpoints that hypertension, and subclinical atherosclerosis in later life originate from blood pressure in childhood [15, 16]. Moreover, longitudinal changes in blood pressure are associated with a greater risk of subclinical renal damage and CKD progression [17, 18]. Thus, the impact of long-term exposure to blood pressure on albuminuria and their correlation needs to be further elucidated. However, data are limited regarding the relationship between cumulative systolic blood pressure (SBP) from childhood and albuminuria at mid-age. In addition, the association of the cumulative phenomenon of SBP from earlier life and albuminuria in later life has not been explored, especially between sexes.

In this study, we aimed to identify subgroups of populations with different cumulative exposure of SBP development from childhood to adulthood and to reveal the relationship of cumulative SBP with albuminuria in midlife, as well as to explore sex differences concerning this relationship.

## Materials and methods

### Study participants

We used the Hanzhong Adolescent Hypertension Cohort, which is a 30-year observational, prospective, population-based cohort study. In this study, 4,623 school students from 26 rural sites in Hanzhong city, Shaanxi, China, were investigated for blood pressure, height, weight and other baselines variables in 1987 to establish the “Hanzhong Adolescent Hypertension Cohort”. The cohort was followed up in 1989, 1992, 1995, 2005, 2013 and 2017, and the response rates were 77.7% (n = 3,592) in 1989, 84.8% (n = 3,918) in 1992, 82.1% (n = 3,794) in 1995, 65.3% (n = 3,018) in 2013, and 60.1% (n = 2,780) in 2017. We randomly selected every tenth participant (K = 10) from the large cohort using an isometric sampling method in 2005 and obtained blood pressure and other data from 436 individuals. Except for the visit in 2005, other follow-ups were large in scale and aimed to visit each individual who was enrolled in 1987. Reasons for loss to follow-up mainly included death, mental illness, military service and migration. The details of these data have been previously published [17, 19]. The flow chart of the cohort study was shown in Additional file 1.

This study was approved by the Ethics Committee of the First Affiliated Hospital of Xi’an Jiaotong University (XJTU1AF2015LSL-047) and was clinically registered (ClinicalTrials.gov. registration number: NCT02734472). The protocol adhered to the principles of the Declaration of Helsinki and all of the participants signed informed consent for each visit. For those individuals < 18 years of age at baseline, consent from a parent/guardian was obtained.

### Data collection

A questionnaire was used to collect personal information, such as demographic characteristics, personal or family medical histories (including hypertension, diabetes and hyperlipidaemia), smoking status, drinking status, and physical activity. Height and weight were measured repeatedly by trained staff by using appropriate scales, and the mean value was used for the data analysis. Moreover, blood pressure was measured three times via a mercury sphygmomanometer in a quiet and comfortable environment (with a 2-minute interval between measurements) by trained and certified staff following WHO-recommended procedures. Participants were required to avoid smoking, alcohol, coffee/tea and vigorous exercise for at least 30 min before blood pressure was measured. The rationale and methodology have been previously described [19].

### Blood and urine biochemical parameters

Blood samples were obtained by peripheral venous puncture, immediately centrifuged at 3000 × g for 10 min, and then stored at − 80℃ until analysis. Total cholesterol, triglyceride, high-density lipoprotein (HDL), low-density lipoprotein (LDL), serum creatinine and blood glucose were measured using an automatic biochemical analyzer (Hitachi, Ltd., Tokyo, Japan), which were measured as described previously [20]. In addition, a morning fasting midstream urine sample was collected from each participant and frozen at − 40℃. All urine samples were shipped in ambient packaging with the use of ice boxes to the clinical laboratory at the First Affiliated Hospital of Xi’an Jiaotong University in Xi’an, China. [21].

### Definitions

Participants who reported continuous or cumulative incidences of smoking for 6 months or more were defined as being smokers [22]. Alcohol consumption was defined as the daily drinking of alcohol (spirits, beer or wine) for 6 months [23]. Physical inactivity was defined as having mild to moderate physical activity < 3 h per week [24]. Moreover, hypertension was defined as an average systolic blood pressure (SBP) of at least 140 mmHg and/or a diastolic blood pressure (DBP) of at least 90 mmHg or the current use of antihypertensive drugs [25]. Diabetes was defined as fasting blood glucose ≥ 7.0 mmol/L or a previous diagnosis at a secondary hospital [26]. Furthermore, hyperlipidaemia was defined as the occurrence of any one of the following situations: hypertriglyceridaemia (TG ≥ 2.26 mmol/L), hypercholesterolemia (TC ≥ 6.22 mmol/L), high levels of LDL-C (≥ 4.14 mmol/L) or low levels of HDL-C (< 1.04 mmol/L) [27] eGFR = 175 × serum creatinine^− 1.234^ × age^− 0.179^ (×0.79 for girls/women), where serum creatinine concentration is in milligrams per deciliter and age is in years [28]. Finally, albuminuria was defined as a urine albumin-to-creatinine ratio (uACR) ≥ 30 mg/g [29, 30].

### Statistical analyses

**Fig. 1:**
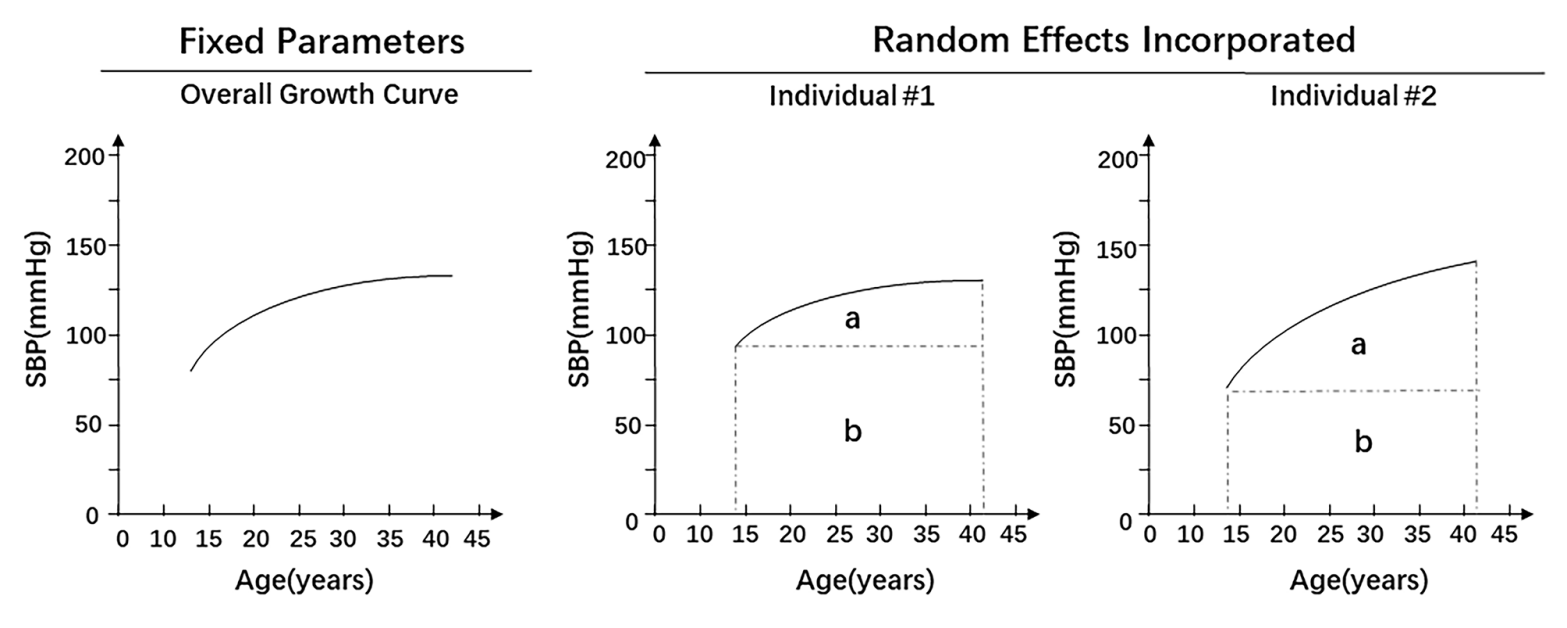
Illustration of the area under the curve (AUC) of systolic blood pressure (SBP). The area under the curve (AUC) of systolic blood pressure (SBP) was calculated as the integral of the curve parameters during the follow-up period in each of these two subjects. The incremental AUCs were different in the two individuals as examples. a = incremental AUC; b = baseline AUC; a + b = total AUC.

Growth curves of SBP, which was measured repeatedly at multiple time points from childhood to adulthood, were constructed by using a random-effects model. The cumulative systolic blood pressure was measured as the area under the curve (AUC) [31] The AUC was calculated as the integral of the curve parameters during the follow-up period for each subject, and the AUC values were divided by the number of follow-up years. As seen in Fig. 1, total AUC (a + b) can be considered a measure of a long-term cumulative burden; incremental AUC (a), which was determined by within-subject variability, represents a combination of linear and nonlinear longitudinal trends, as we used in our previous studies [32]. To fit the AUC, we included subjects with four or more SBP measurements and excluded those individuals with data form less than two of the first four visits or form the last three visits (at least 2 times each in youth and middle-aged). As a result, a total of 1,683 individuals were included in the SBP curve construction No significant difference was observed between those who were followed and lost to follow-up (see Additional file 2). To exclude the potential influence of antihypertensive, hypoglycaemic or lipid-lowering medications, we performed sensitivity analyses.

Continuous data were expressed as means ± SDs, otherwise, they were presented as median (25th, and 75th percentile ranges). Categorical variables are expressed as frequencies and percentages. Continuous variables between the two groups were compared using the t test or Mann–Whitney U test according to the normality of the distribution, and one-way ANOVA or Kruskal–Wallis tests were used for three or more groups. Moreover, we divided participants into low, middle and high groups according to the tertiles of total AUC and incremental AUC. A multiple logistic regression analysis was used to determine the association between albuminuria in 2017 and different cumulative SBP groups. P values of logistic regression were adjusted with Benjamini and Hochberg (false discovery rate) analysis in the revised manuscript by R software (padjust Method = “BH”) to decreases of false positive finding. Sex differences are expressed as P values of the interaction terms, which were adjusted for the covariates. The statistical analysis was performed by using SPSS 23.0 software (SPSS Inc., Chicago, Illinois, USA) and Python 3.8. A P value less than 0.05 was considered to be statistically significant in this study.

## Results

### Characteristics of the study population

The study population included of 1,683 individuals for cumulative SBP measurements from childhood to middle age. Baseline characteristics in 1987 for men and women are shown in Table 1. Males accounted for 978 (58.1%) and women accounted for 705 (41.9%) of the study population. Men tended to have higher smoking incidence, alcohol consumption, BMI, SBP, DBP, blood glucose, total cholesterol, triglycerides, LDL, SUA, serum creatinine, urine creatinine and urine microalbumin levels, as well as the prevalence of hypertension, diabetes and hyperuricaemia but lower HDL, eGFR and uACR values, in middle age. Males had a higher SBP total AUC (122.5 mmHg for males vs. 114.2 mmHg for females, *P* < 0.001) and incremental AUC (18.5 mmHg for males vs. 7.9 mmHg for females, *P* < 0.001) than females. The levels of uACR in the male and female group was 8.0 (5.4–14.0) and 10.2 (6.4–17.7), respectively (P for trend < 0.001). Furthermore, there were no significant sex differences in height, weight, BMI, SBP at baseline (P > 0.05) or age in 2017 (P for trend = 0.503).

**Table 1:** Demographic and clinical characteristics of the study participants at baseline and the latest follow-up, by sex

Characteristics	ALL	Male	Female	*P* value
No. of subjects	1683	978	705	
**Childhood (Baseline in 1987)**				
Age (years)	12.0 (9.0–14.0)	12.0 (9.0–14.0)	12.0 (9.0–14.0)	0.814
Height (cm)	139.0 (125.0-151.5)	138.2 (124.6–153.0)	140.7 (125.0-150.5)	0.436
Weight (kg)	31.0 (23.3–41.9)	30.5 (23.4–41.9)	32.5 (23.1–41.9)	0.725
BMI (kg/m^2^)	16.17 (14.88–18.12)	16.1 (15.0-17.9)	16.3 (14.7–18.5)	0.290
SBP (mmHg)	104.0 (97.3-111.3)	103.3 (96.7–112.0)	104.7 (98.0-110.7)	0.569
DBP (mmHg)	64.7 (60.0-71.3)	64.0 (59.3–71.3)	66.0 (60.0-71.3)	0.029
**Adulthood (Latest follow-up in 2017)**				
Age (years)	44.0 (41.0–46.0)	44.0 (41.0–46.0)	44.0 (41.0–46.0)	0.503
Current smoking (n, %)	768 (45.6%)	742 (75.0%)	26 (3.7%)	< 0.001
Alcohol consumption (n, %)	509 (30.2%)	465 (47.5%)	44 (6.2%)	< 0.001
Exercise (n, %)	465 (27.6%)	223 (22.8%)	242 (34.3%)	< 0.001
Hypertension (n, %)	203 (12.1%)	153 (15.6%)	50 (7.1%)	< 0.001
Diabetes mellitus (n, %)	55 (3.3%)	38 (3.9%)	17 (2.4%)	0.093
Hyperlipidaemia (n, %)	172 (10.2%)	126 (12.9%)	46 (6.5%)	< 0.001
BMI (kg/m^2^)	23.9 (21.9-26.41)	24.5 (22.4–36.5)	23.1 (21.5–25.2)	< 0.001
Waist (cm)	84.7 (78.2–91.7)	88.1 (81.6–94.0)	80.7 (75.6–86.9)	< 0.001
Hips (cm)	92.1 (88.8–95.6)	92.6 (89.4–96.2)	91.4 (88.2–95.2)	< 0.001
SBP (mmHg)	121.7 (112.7-131.7)	125.0 (117.0-134.3)	116.3 (108.3-126.3)	< 0.001
DBP (mmHg)	76.3 (69.3–84.3)	79.3 (72.7–86.7)	72.3 (65.7–80.0)	< 0.001
GLU (mmol/L)	4.58 (4.3–4.9)	4.60 (4.3–5.9)	4.50 (4.3–4.8)	0.003
ALT (U/L)	19.0 (14.0–28.0)	24.0 (17.0–34.0)	15.0 (11.0–19.0)	< 0.001
AST (U/L)	16.0 (13.0–21.0)	18.0 (14.0–23.0)	14.0 (12.0–18.0)	< 0.001
Total cholesterol (mmol/L)	4.5 (4.0–5.0)	4.5 (4.1–5.1)	4.5 (4.0–5.0)	0.006
Triglycerides (mmol/L)	1.4 (1.0–2.0)	1.5 (1.1–2.2)	1.2 (0.9–1.7)	< 0.001
LDL (mmol/L)	2.5 (2.1–2.9)	2.6 (2.2-3.0)	2.4 (2.1–2.8)	< 0.001
HDL (mmol/L)	1.1 (1.0-1.3)	1.1 (0.9–1.2)	1.2 (1.1–1.4)	< 0.001
SUA (µmol /L)	283.5 (228.0-338.9)	323.5 (280.7-367.3)	226.6 (197.1-268.9)	< 0.001
Serum creatinine (µmol/L)	76.5 (67.6–86.6)	83.7 (75.9–91.3)	67.6 (60.8–74.0)	< 0.001
Urine creatinine (µmol/L)	7885.0 (4480.0-13023.0)	9309.0 (5570.0-14681.5)	6078.0 (3447.0-10594.5)	< 0.001
eGFR (mL/min/1.73m^2^)	96.3 (86.4-109.5)	95.2 (85.7-107.9)	98.1 (87.5–112.0)	0.001
mALB (mg/L)	8.0 (4.2–14.2)	8.9 (4.8–15.5)	6.5 (3.6–12.8)	< 0.001
uACR (mg/g)	8.8(5.7–15.5)	8.0(5.4–14.0)	10.2(6.4–17.7)	< 0.001
SBP total AUC (mmHg)	118.8 (111.2-127.2)	122.5 (114.7-130.2)	114.2 (106.9–121.0)	< 0.001
SBP incremental AUC (mmHg)	14.2 (7.9–23.2)	18.5 (9.9–26.1)	7.9 (1.1–16.6)	< 0.001
DBP total AUC (mmHg)	75.8 (70.1–81.4)	77.8 (71.8–83.2)	72.7 (68.3–78.5)	< 0.001
DBP incremental AUC (mmHg)	10.7 (4.0-17.9)	13.7 (6.2–20.6)	7.5 (1.8–14.0)	< 0.001

Continuous variables were shown as mean ± SD if normally distributed or median (quartile 1, quartile 3) if non-normally distributed. Categorical variables were expressed as numbers and percentages of subjects. The Mann–Whitney test was used for non-normally distributed continuous variables. Differences between groups of categorical variables were compared with chi-squared tests. BMI, body mass index; SBP, systolic blood pressure; DBP, diastolic blood pressure; GLU, fasting plasma blood glucose; ALT, alanine transaminase; AST, aspartate-aminotransferase; LDL, low-density lipoprotein; HDL, high-density lipoprotein; SUA, serum uric acid; eGFR, estimated glomerular filtration rate; mALB, Urine albumin; uACR, urinary albumin-to-creatinine ratio; AUC, area under the curve

### UACR and albuminuria among different SBP AUC groups

**Fig. 2:**
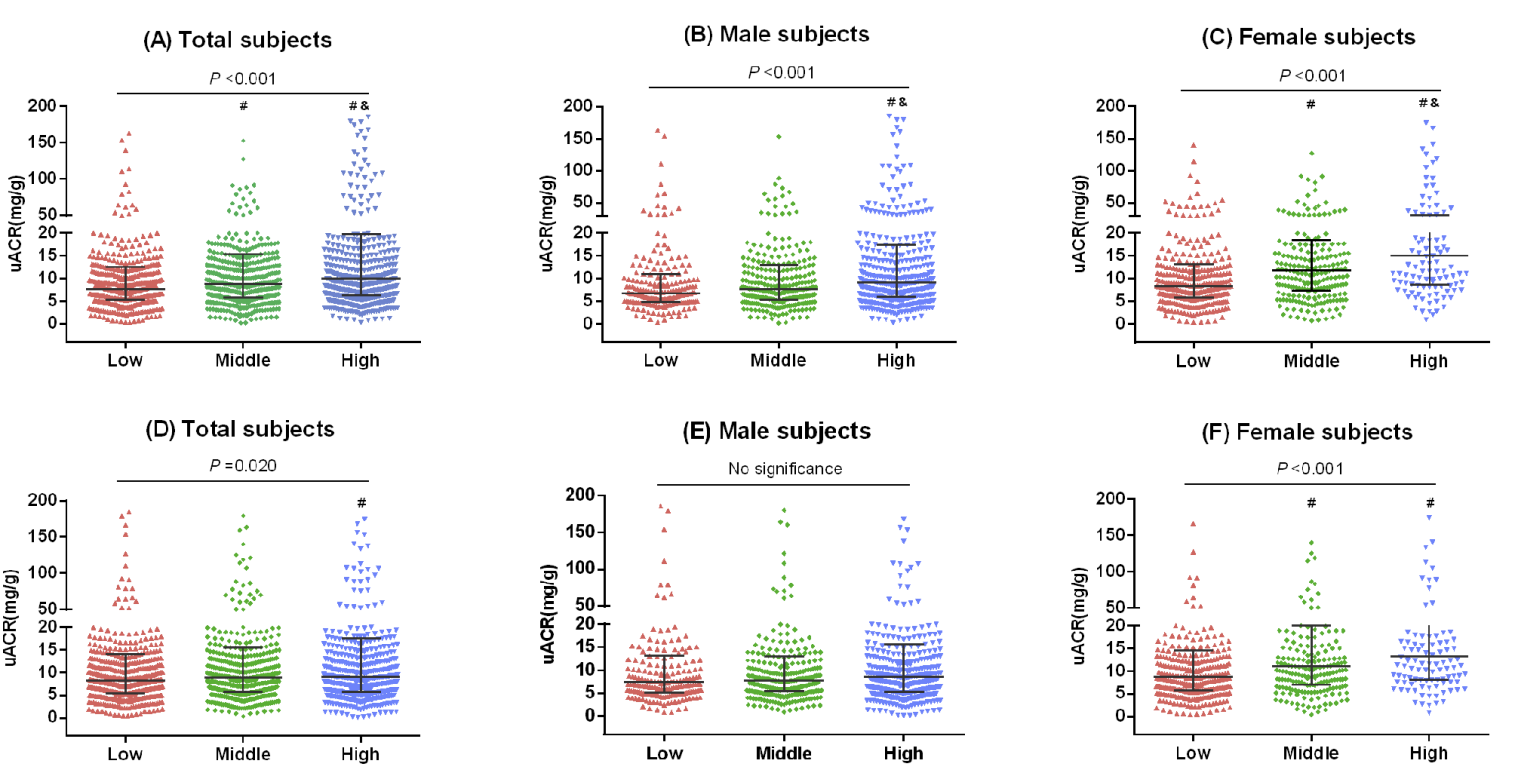
The urinary albumin-to-creatinine ratio (uACR) by tertiles of cumulative SBP. **(A)** Total AUC of SBP in all of the subjects, **(B)** total AUC of SBP in male subjects, **(C)** total AUC of SBP in female subjects; **(D)** incremental AUC of SBP in all of the subjects, **(E)** incremental AUC of SBP in male subjects, **(F)** incremental AUC of SBP in female subjects. Low, the first tertile of cumulative SBP; Middle, the second tertile; High, the third tertile. The uACR values among different groups were compared with the Kruskal-Wallis test; ^#^: *P* < 0.05 compared with the Low group; ^&^: *P* < 0.05 compared with the Middle group

In all of the individuals, the middle- and high-AUC groups of SBP had a higher incidence of albuminuria than the low-AUC group, and other demographic and clinical characteristics of the participants by AUC group are shown in more detail [see Additional file 2 and 3]. Figure 2 presents the levels of uACR by tertiles of cumulative SBP in terms of total AUC values and incremental AUC values. The uACR values exhibited increased trends as the total AUC increased: 7.71 (5.32–12.49) in the low total AUC group, 8.83 (5.76–15.27) in the middle total AUC group and 9.98 (6.27–19.70) in the high total AUC group (P for trend = 0.006 after adjusting age and sex) (Fig. 2A). Similar results were observed across the incremental AUC groups (P for trend = 0.042 after adjusting age and sex) (Fig. 2D). Interestingly, the uACR levels were significantly higher in the middle and high groups than in the low group in female subjects (P for trend < 0.05 after adjusting age) (Fig. 2C F), and differences in uACR values in females are more obvious than males (Fig. 2E F).

**Fig. 3:**
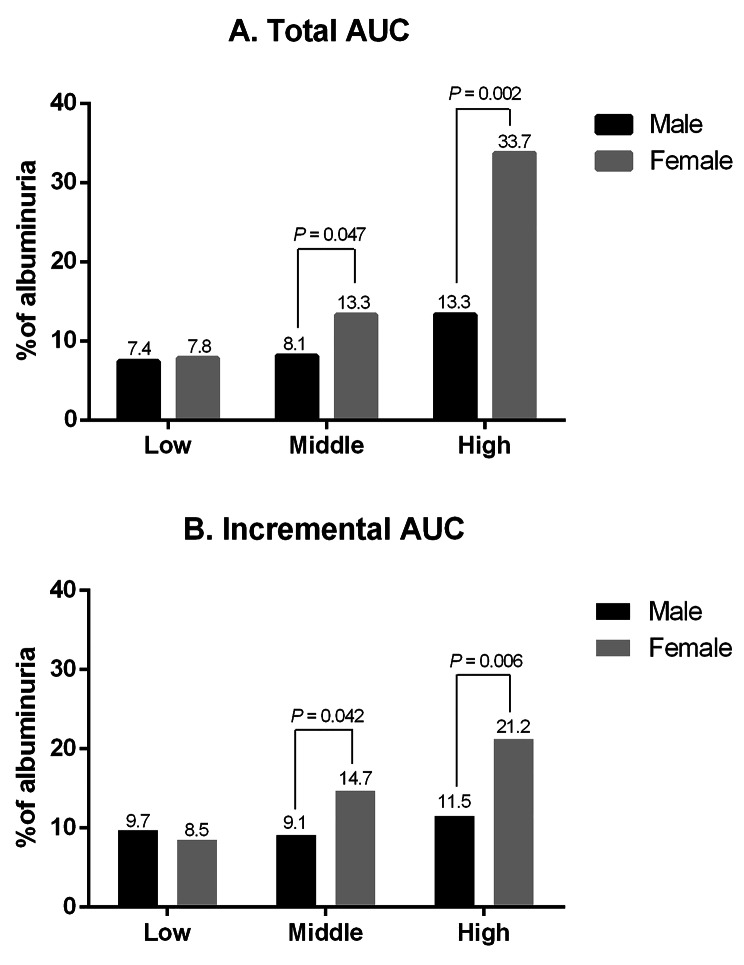
Differences in the prevalence of albuminuria between sexes by cumulative SBP: **(A)** total AUC of SBP; **(B)** incremental AUC of SBP

Over 30 years of follow-up, a total of 190 people developed albuminuria, including 53.2% males. Figure 3 shows that the incidence of albuminuria in middle age varied across the total AUC and incremental AUC groups: 33.7% albuminuria for females and 13.3% for males in the high total AUC group (*P* = 0.002), and 21.2% for females and 11.5% for males in the high incremental AUC group (*P* = 0.006). Women exhibited a higher albuminuria incidence in the middle and high SBP AUC groups than men did.

### Association of cumulative SBP with albuminuria in middle age by sex

The results from the logistic regression models for examining the association between total AUC of SBP and albuminuria are summarized in **Table 2**. Different total AUC groups of SBP were included as independent variables in a logistic regression model, and the low group was considered the control group. In the unadjusted model, the ORs (95% CIs) of albuminuria were 1.11 (0.58–2.11) for the middle group and 1.93 (1.08–3.43) for the high group in the male subjects; additionally, they were 1.80 (1.05–3.10) for the middle group and 3.97 (2.24–7.02) for the high group in female subjects. After adjusting for demographic characteristics (age, sex), anthropometric parameters (BMI, waist and hip measurements), life and disease history (smoking status, alcohol consumption, exercise, hypertension, diabetes and hyperlipidaemia in 2017), the ORs decreased (Table 2, Model 1). Additional adjustments were performed with individual biochemical indicators in 2017, and the ORs of albuminuria for males and females in the high group were 1.57 (0.77–3.23) and 2.68 (1.30–5.53), respectively. Females tended to show an increased risk of albuminuria according to their total AUC groups (P for trend = 0.007). **Table 3** shows the results for the logistic regression models examining the association between incremental AUC and adult albuminuria. The SBP incremental AUC for females was associated with adult albuminuria, whereas that for males was not. After adjustments for multiple confounders, compared with the low group, the ORs of albuminuria for females were 1.83 (1.01–3.33) in the middle group and 2.34 (1.14–4.79) in the high group. There was a significant interaction between total AUC and sex on the outcomes of albuminuria in all of the models (P < 0.001 for interaction), which suggested that higher total AUC had a more detrimental effect in women. Moreover, the interaction terms of incremental AUC and sex also obtained statistical significance for adult albuminuria (P for trend = 0.040). Particularly, we analyzed the relationship between cumulative DBP and albuminuria. As presented in Additional file 15 and 6, after adjustments for multiple confounders, compared with the low group, total and incremental AUC values of DBP showed no significant associations with the incident albuminuria by middle age, except for this relationship in total AUC of DBP among total subjects.

**Table 2:** Association of SBP total AUC groups with midlife albuminuria by sex

**Characteristics**	**N (%)**	**Unadjusted**		**Model 1**		**Model 2**		
		**OR (95%CI)**	***P*** **value**	**OR (95%CI)**	***P*** **value**	**OR (95%CI)**	***P*** **value**	P*
**Total subjects**								< 0.001
Low	43 (7.7%)	1.00	-	1.00	-	1.00		
Middle	58 (10.3%)	1.39 (0.92–2.10)	0.119	1.39 (0.89–2.16)	0.145	1.30 (0.82–2.06)	0.261	
High	89 (15.9%)	2.27 (1.55–3.33)	< 0.001^#^	1.92 (1.18–3.11)	0.008^#^	1.92 (1.18–3.15)	0.009^#^	
**Male Subjects**								
Low	16 (7.4%)	1.00	-	1.00	-	1.00	-	
Middle	26 (8.1%)	1.11 (0.58–2.11)	0.762	1.08 (0.54–2.12)	0.835	1.17 (0.56–2.46)	0.672	
High	59 (13.3%)	1.93 (1.08–3.43)	0.026^#^	1.28 (0.66–2.51)	0.465	1.57 (0.77–3.23)	0.218	
**Female Subjects**								
Low	27 (7.8%)	1.00	-	1.00	-	1.00	-	
Middle	32 (13.3%)	1.80 (1.05–3.10)	0.033	1.65 (0.91–3.98)	0.097	1.50 (0.82–2.76)	0.188	
High	30 (33.7%)	3.97 (2.24–7.02)	< 0.001^#^	2.95 (1.47–5.93)	0.002^#^	2.68 (1.30–5.53)	0.007^#^	

Model 1: adjusted for age, gender, systolic blood pressure, body mass index at baseline, body mass index, waist, hips, smoking, alcohol consumption, exercise, and history of hypertension, diabetes, hyperlipidaemia in 2017.

Model 2: adjusted for model 1 + fasting blood glucose, ALT, AST, total cholesterol, triglycerides, low-density lipoprotein, high-density lipoprotein, serum uric acid and serum creatinine.

P*: p value for interaction terms of total AUC and sex.

^#^: *p* value was considered significant after false discovery rate correction.

**Table 3:** Association of SBP incremental AUC groups with midlife albuminuria by sex

**Characteristics**	**N (%)**	**Unadjusted**		**Model 1**		**Model 2**		
		**OR(95%CI)**	***P*** **value**	**OR(95%CI)**	***P*** **value**	**OR(95%CI)**	***P*** **value**	P*
**Total subjects**								
Low	50 (8.9%)	1.00	-	1.00	-	1.00	-	0.040
Middle	64 (11.4%)	1.32 (0.89–1.94)	0.168	1.42 (0.92–2.18)	0.112	1.48 (0.94–2.31)	0.089	
High	76 (13.5%)	1.60 (1.10–2.34)	0.015^#^	1.52 (0.94–2.45)	0.087	1.60 (0.98–2.62)	0.062	
**Male Subjects**								
Low	20 (9.7%)	1.00	-	1.00	-	1.00	-	
Middle	30 (9.1%)	0.93 (0.52–1.69)	0.820	0.87 (0.45–1.66)	0.665	1.00 (0.50–2.02)	0.992	
High	51 (11.5%)	1.21 (0.70–2.09)	0.494	0.90 (0.47–1.75)	0.764	1.08 (0.54–2.17)	0.829	
**Female Subjects**								
Low	30 (8.5%)	1.00	-	1.00	-	1.00	-	
Middle	34 (14.7%)	1.86 (1.10–3.14)	0.020	1.90 (1.07–3.40)	0.029	1.83 (1.01–3.33)	0.046	
High	25 (21.2%)	2.91 (1.63–5.19)	< 0.001^#^	2.52 (1.25–5.07)	0.010^#^	2.34 (1.14–4.79)	0.020^#^	

Model 1: adjusted for age, gender, systolic blood pressure, body mass index at baseline, body mass index, waist, hips, smoking, alcohol consumption, exercise, and history of hypertension, diabetes, hyperlipidaemia in 2017.

Model 2: adjusted for model 1 + fasting blood glucose, ALT, AST, total cholesterol, triglycerides, low-density lipoprotein, high-density lipoprotein, serum uric acid and serum creatinine.

P*: p value for interaction terms of incremental AUC and sex.

^#^: *p* value was considered significant after false discovery rate correction.

### Sensitivity analyses

**Fig. 4:**
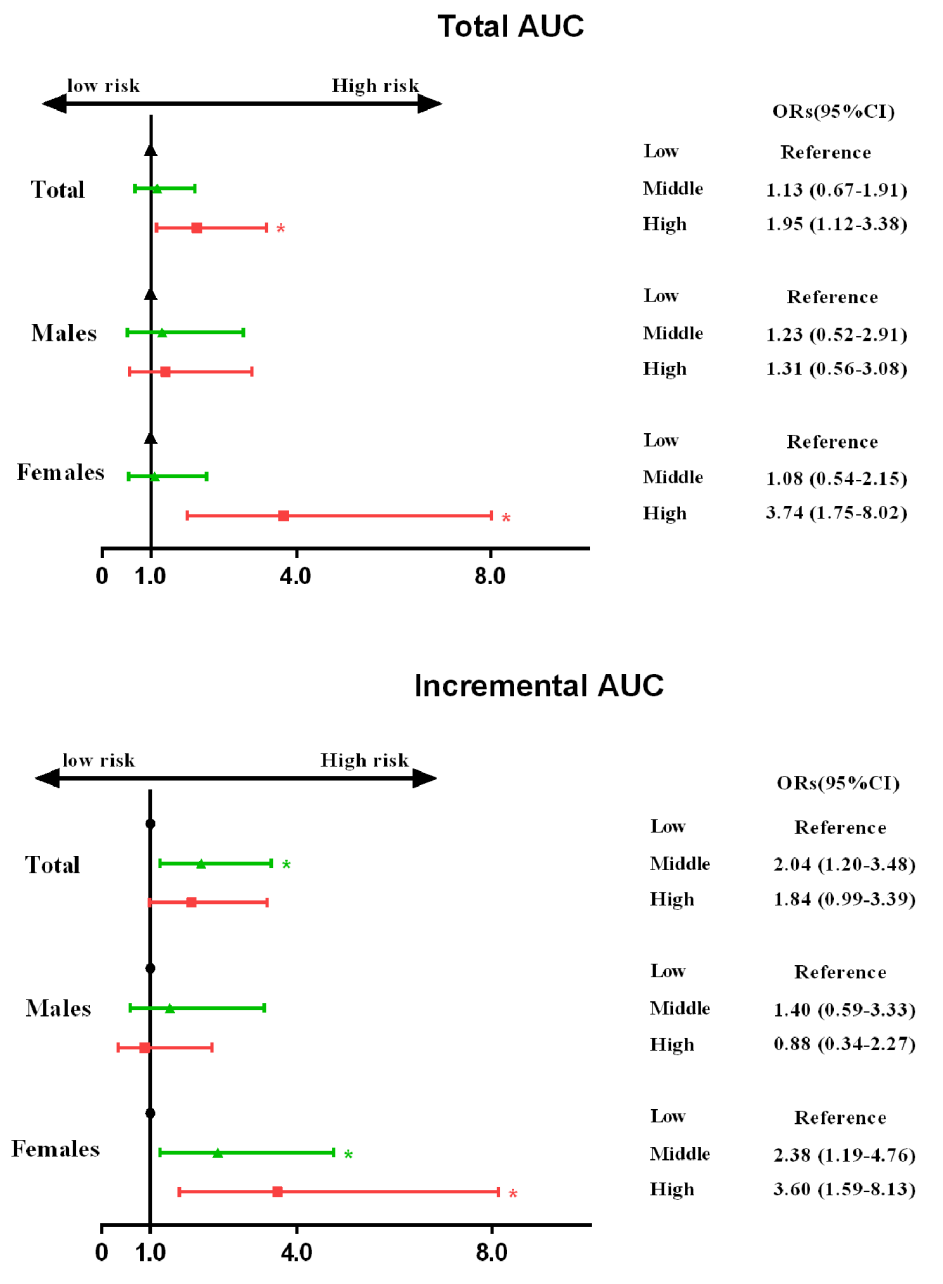
Effects of the cumulative burden and trends of SBP (classified according to tertiles) on albuminuria in adults between sexes(sensitivity analyses). All models adjusted for age, sex, body mass index at baseline, body mass index, waist, hips, smoking statue, alcohol consumption, exercise, history of hypertension, diabetes, hyperlipidaemia in 2017, and fasting blood glucose, ALT, AST, total cholesterol, triglycerides, low-density lipoprotein, high-density lipoprotein, serum uric acid and serum creatinine

We further excluded patients with hypertension, diabetes, or hyperlipidaemia (n = 320) for further analysis to eliminate the effects of these disease states on the results. Multivariate-adjusted ORs for sensitivity by sex are shown in Fig. 4. Similar results of cumulative SBP on the risk of midlife albuminuria by different AUC groups existed in males and females. It should be considered that an increased risk of albuminuria with incremental AUC in women was observed: 2.38 (1.19–4.76) for the middle group and 3.60 (1.59–8.13) for the high group. Moreover, the interaction effects of SBP AUC and sex were significant (P = 0.012 for sex with total AUC; P = 0.021 for sex with incremental AUC).

## Discussion

In this study, we first investigated the sex difference in the relationship of cumulative SBP from childhood to adulthood with albuminuria in midlife. Growth curves of cumulative SBP over a 30-year follow-up were constructed to assess its effect on albuminuria. We identified a significant relationship between long-term cumulative SBP burden and sex on adult albuminuria that women with a higher total AUC showed a tendency for elevated risk compared with men, and longitudinal trends also exhibited a sex difference. Our findings suggest that the determination of sex-specific cumulative SBP from childhood to adulthood may provide a viable source for distinguishing populations with an increased risk for developing albuminuria.

High blood pressure is an important risk factor in the development of a series of cardiovascular and albuminuria disorders, and albuminuria has been considered to be a sign of hypertensive renal damage, as well as a forerunner to renal and cardiovascular disease [6, 33]. The relationship between blood pressure and albuminuria is complicated and bidirectional, as hypertension can lead to worsened proteinuria resulting in renal damage and urinary albumin excretion rates associated with BP progression and hypertension incidence [33–35]. Considerable research efforts have been devoted to the relationship between blood pressure and albuminuria. Eric et al [13] observed that high-normal blood pressure was significantly associated with increased odds of microalbuminuria, compared with optimal blood pressure. Additionally, a cross-sectional study revealed that higher blood pressure was strongly associated with albuminuria among individuals with or without diabetes and marked atherosclerosis [14]. In addition, intensive blood pressure management has been shown to the risk of major cardiovascular events and the development of albuminuria in patients with diabetes [10]. Apparently, it is almost indisputable that blood pressure is an important risk factor for albuminuria.

Studies have demonstrated that high blood pressure in adults originates from childhood, and elevated blood pressure in childhood increases the risk of developing high blood pressure in adults [36]. Several anthropometric variables including blood pressure, height, body weight and body mass index, always changes with growth and age; therefore, the longitudinal observation of these changes plays a significant role in clinical research. Numerous studies have confirmed that cumulative systolic blood pressure is associated with disease or dysfunction in adulthood [37, 38]. In the Multi-Ethnic Study of Atherosclerosis (MESA) initiative, Zemaitis et al [39] reported that incidences of higher cumulative SBP was associated with increased UACR progression in adults with a 10-year follow-up. In line with previous studies, our result suggested that higher cumulative exposure to blood pressure is associated with a risk of developing albuminuria in middle-life. Moreover, the uniqueness of this study results from the cumulative SBP that originates from childhood, as well as experiences of puberty, adulthood, and early middle age, which focus on cumulative blood pressure over a 30-year period. A further novel finding is that, the positive association between longitudinal cumulative SBP and albuminuria was evident for women but not for men, which means we should likely focus more attention to the long-term accumulation and increasing trend of SBP in women. Albuminuria is strongly associated with several diseases or life states, such as diabetes, hypertension and obesity [40–42]. Therefore, we excluded people with diabetes, hypertension or hyperlipidaemia and adjusted for other variables for the sensitivity analysis, and the finding was similar to the conclusions stated above. Our observations suggest that the control of cumulative SBP from childhood for maintaining a relatively lower level is essential for the prevention of albuminuria and more cardiovascular and renal diseases.

There is a strong association between blood pressure and the risk of albuminuria; however, the results on sex differences are rare and inconsistent. Joel et al. [43] reported that men with chronic kidney disease of various etiologies experienced a faster rate of decline in renal function over time than women. In a study of 223 nondiabetic patients diagnosed with mild hypertension, male sex and 24-hour systolic blood pressure were significant determinants of microalbuminuria [44]. However, these studies on the association between blood pressure and albuminuria are based on data from a single blood pressure measurement, multiple blood pressure measurements over a short time or 24-hour ambulatory blood pressure measurements. In contrast, a global-based review reported that in most regions of the world, the prevalence of CKD among women is higher than in men [45]. Albuminuria is a well-recognized marker of microcirculatory damage that is more applicable in young and middle-aged adults,[46] and proteinuria should be considered a predictor of a decline in glomerular filtration rate and the development of end-stage renal disease [47]. We speculate that this may be due to albuminuria being an early marker of kidney damage and sex differences appearing before the onset of chronic kidney disease. This may explain why higher cumulative SBP is more significantly associated with albuminuria in women. In addition, we found that the prevalence of smoking in men was very high. In order to rule out the impact of smoking on gender differences, we analyzed the relationship between smoking status and albuminuria in this study (Additional file 7). The results showed that there was no significant correlation between smoking status and adult albuminuria. Indeed, we propose that more clinical evidence and mechanisms of how cumulative SBP affects albuminuria by sex should be clarified in future studies.

Our current study had several strengths. This prospective cohort with a regular follow-up period of 30 years included children and adolescents at baseline and provides opportunity to demonstrate a significant inverse correlation between early-life SBP cumulative exposure and albuminuria in midlife. Moreover, we used the long-term cumulative burden and trend of BP (measured as total AUC and incremental AUC, respectively) to predict the occurrence of albuminuria in middle-aged adults of different sexes. However, our analysis had several limitations. For example, most of our participants were from the Han nationality in rural areas of northern China. Nevertheless, it also reduces potential confounding factors caused by race and living environments. In addition, albuminuria was assessed with spot morning urine samples and not 24-hour urine collection. However, our spot morning urine samples are collected, transported, and stored in a strict way by professional physicians; additionally, in some large clinical studies, spot urine is also used instead of 24-hour urine to evaluate proteinuria [28, 48]. What’s more, participants with median age of 44 years old in the last follow-up experienced a relatively low prevalence of kidney disease; and the number of participants with albuminuria was rather small.

In summary, we demonstrated that a higher cumulative burden and trends of SBP with a 30-year follow-up was independently associated with uACR levels and the incidence of proteinuria in midlife. Furthermore, we have shown for the first time that this association was more apparent in female individuals, but not in males. These results underscore the importance of controlling cumulative SBP levels to prevent albuminuria in adult life, especially in women.

## Electronic supplementary material

Below is the link to the electronic supplementary material.

Supplementary Material 1

Supplementary Material 2

Supplementary Material 3

Supplementary Material 4

Supplementary Material 5

Supplementary Material 6

Supplementary Material 7

## Data Availability

The study includes no data from published or unpublished human clinical databases. The data that support the findings of this study are available from the corresponding author upon reasonable request.
