# Supplementary material for: Sex differences in impact of cumulative systolic blood pressure from childhood to adulthood on albuminuria in midlife: a 30-year prospective cohort study
Source: BMC Public Health. 2023 Apr 11;23:666. doi: 10.1186/s12889-023-15613-y (PMC10088136; doi:10.1186/s12889-023-15613-y)
Supplement: Supplementary file 1 — Supplementary Material 1 [file 12889_2023_15613_MOESM1_ESM.docx]

Additional file 1. Demographic and clinical characteristics categorized by those who were included and excluded in the study and those who were lost follow-up

| **Characteristics** | **Group A (n=1843)** | **Group B (n=1097)** | **Group C (n=1683)** | ***P* value** |
| --- | --- | --- | --- | --- |
| **Childhood (Baseline in 1987)** | | | | |
| Age in 1987 (years) | 14.0 (12.0-16.0) | 13.0 (9.0-16.0) | 12.0 (9.0-14.0) | 0.246 |
| BMI in 1987 (kg/m^2^) | 16.2 (14.5-18.2) | 16.3 (14.9-18.3) | 16.2 (14.8-18.1) | 0.224 |
| SBP in 1987 (mmHg) | 105.3 (98.0-112.0) | 103.3 (97.3-110.7) | 104.0 (97.3-111.3) | 0.253 |
| DBP in 1987 (mmHg) | 64.7 (59.3-71.3) | 64.7 (59.3-70.7) | 64.7 (60.0-71.3) | 0.481 |
| Males (n, %) | 964 (53.3%) | 609 (55.5%) | 987 (58.7%) | 0.104 |
| BMI in 2017 (kg/m^2^) | - | 23.6 (21.8-25.9) | 23.9 (21.9-26.41) | 0.214 |
| Waist in 2017(cm) | - | 84.6 (78.3-91.7) | 84.7 (78.2-91.7) | 0.603 |
| Hips in 2017 (cm) | - | 92.0 (88.5-95.5) | 92.1 (88.8-95.6) | 0.407 |
| Current smoking (n, %) | - | 390 (35.5%) | 768 (45.6%) | <0.001 |
| Alcohol consumption (n, %) | - | 281 (25.6%) | 509 (30.2%) | 0.069 |
| Exercise (n, %) | - | 312 (28.4%) | 465 (27.6%) | 0.513 |
| Hypertension (n, %) | - | 103 (9.4%) | 203 (12.1%) | 0.286 |
| Diabetes mellitus (n, %) | - | 23 (2.7%) | 55 (3.3%) | 0.088 |
| Hyperlipidaemia (n, %) | - | 99 (9.0%) | 172 (10.2%) | 0.070 |
| SBP in 2017 (mmHg) | - | 120.7 (112.6-131.0) | 121.7 (112.7-131.7) | 0.587 |
| DBP in 2017 (mmHg) | - | 76.0 (69.3-83.7) | 76.3 (69.3-84.3) | 0.395 |
| GLU (mmol/L) | - | 4.5 (4.2-4.9) | 4.6 (4.3-4.9) | 0.092 |
| Total cholesterol (mmol/L) | - | 4.5 (4.1-5.0) | 4.5 (4.0-5.0) | 0.749 |
| Triglycerides (mmol/L) | - | 1.4 (1.0-1.8) | 1.4 (1.0-2.0) | 0.638 |
| LDL (mmol/L) | - | 2.5 (2.1-2.9) | 2.5 (2.1-2.9) | 0.946 |
| HDL (mmol/L) | - | 1.15 (1.01-1.35) | 1.14 (0.99-1.33) | 0.106 |
| Serum creatinine (mmol/L) | - | 76.1 (67.3-86.8) | 76.5 (67.6-86.6) | 0.820 |
| eGFR (mL/min/1.73m^2^) | - | 98.3 (87.1-110.8) | 96.3 (86.4-109.5) | 0.121 |
| uACR (mg/g) | - | 8.5 (5.2-14.8) | 8.8 (5.7-15.5) | 0.270 |

Group A: enrolled in the study in 1987 but not seen in 2017, Group B: enrolled in the study and seen in 2017 but not included in the present analysis, Group C: enrolled in the study 2017 and included in the present analysis. BMI, body mass index; SBP, systolic blood pressure; DBP, diastolic blood pressure; GLU, fasting blood glucose; LDL, low-density lipoprotein; HDL, high-density lipoprotein; eGFR, estimated glomerular filtration rate; uACR, urinary albumin-to-creatinine ratio. Non-normally distributed variables are expressed as the median (interquartile range). All other values are expressed as mean ± SD or n, %
