# Supplementary figures and images for: Sex differences in impact of cumulative systolic blood pressure from childhood to adulthood on albuminuria in midlife: a 30-year prospective cohort study
Source: BMC Public Health. 2023 Apr 11;23:666. doi: 10.1186/s12889-023-15613-y (PMC10088136; doi:10.1186/s12889-023-15613-y)

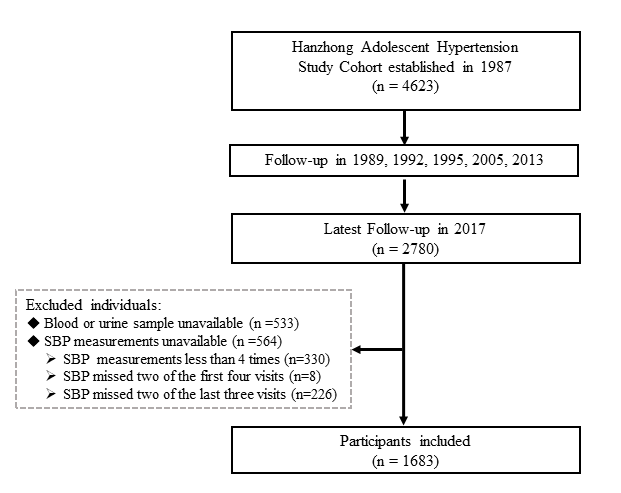


**Additional file 2**. The flow chart of the cohort study

Supplement: Supplementary file 2 — Supplementary Material 2 [file 12889_2023_15613_MOESM2_ESM.docx]
