# Supplementary material for: Sex differences in impact of cumulative systolic blood pressure from childhood to adulthood on albuminuria in midlife: a 30-year prospective cohort study
Source: BMC Public Health. 2023 Apr 11;23:666. doi: 10.1186/s12889-023-15613-y (PMC10088136; doi:10.1186/s12889-023-15613-y)
Supplement: Supplementary file 3 — Supplementary Material 3 [file 12889_2023_15613_MOESM3_ESM.docx]

| **Additional file 3.** Demographic and clinical characteristics of the participants at baseline and the latest follow-up, by total AUC of SBP | | | | |
| --- | --- | --- | --- | --- |
| **Characteristics** | **Low**  **(AUC< 113.82)** | **Middle**  **(113.83≤AUC≤124.04)** | **High**  **(AUC >124.04)** | ***P* value** |
| Male (n, %) | 216 (38.5%) | 320 (57.0%) | 442 (78.8%) | <0.001 |
| n (%) of albuminuria | 43 (7.7%) | 58 (10.3%) | 89 (15.9%) | <0.001 |
| **Childhood** |  |  |  |  |
| Age (years) | 11.0 (8.0-14.0) | 12.0 (9.0-14.0) | 13.0 (11.0-15.0) | <0.001 |
| Height (cm) | 131.0 (119.1-148.4) | 138.0 (126.0-152.5) | 144.4 (132.5-154.4) | <0.001 |
| Weight (kg) | 26.2 (20.4-38.4) | 30.9 (23.5-42.5) | 34.9 (27.4-44.5) | <0.001 |
| BMI (kg/m^2^) | 15.5 (14.4-17.6) | 16.2 (14.9-18.2) | 16.8 (15.5-18.6) | <0.001 |
| SBP (mmHg) | 100.0 (93.3-107.3) | 104.0 (97.0-111.3) | 108.7 (100.7-116.0) | <0.001 |
| DBP (mmHg) | 61.3 (58.0-69.3) | 64.7 (60.0-70.7) | 68.6 (60.8-74.0) | <0.001 |
| **Adulthood** |  |  |  |  |
| Age(years) | 42.0 (40.0-45.0) | 44.0 (41.0-46.0) | 45.0 (42.0-46.0) | <0.001 |
| Current smoking (n, %) | 181 (32.3%) | 252 (44.9%) | 335 (59.7%) | <0.001 |
| Alcohol consumption (n, %) | 121 (21.6%) | 168 (29.9%) | 220 (39.2%) | <0.001 |
| Exercise (n, %) | 164 (29.2%) | 156 (27.8%) | 145 (25.8%) | 0.444 |
| Hypertension (n, %) | 11 (2.0%) | 39 (7.0%) | 153 (27.3%) | <0.001 |
| Diabetes mellitus (n, %) | 8 (1.4%) | 18 (3.2%) | 29 (5.2%) | 0.002 |
| Hyperlipidaemia (n, %) | 37 (6.6%) | 64 (11.4%) | 71 (12.7%) | 0.002 |
| BMI (kg/m2) | 23.0 (21.2-25.2) | 23.7 (22.0-25.8) | 24.9 (22.8-26.8) | <0.001 |
| Waist (cm) | 82.0 (76.0-88.9) | 84.1 (78.1-91.1) | 88.6 (81.8-94.1) | <0.001 |
| Hips (cm) | 91.2 (88.2-94.8) | 91.7 (88.6-95.7) | 93.2 (90.0-96.6) | <0.001 |
| SBP (mmHg) | 113.3 (105.7-121.3) | 121.3 (114.0-129.3) | 131.3 (123.0-143.8) | <0.001 |
| DBP (mmHg) | 70.3 (64.7-76.0) | 76.0 (69.7-82.7) | 84 (76.7-91.0) | <0.001 |
| Fasting glucose (mmol/L) | 4.5 (4.3-4.8) | 4.5 (4.3-4.9) | 4.7 (4.3-5.0) | <0.001 |
| ALT (U/L) | 18.0 (13.0-25.0) | 19.0 (13.0-27.0) | 21.0 (15.5-31.0) | <0.001 |
| AST (U/L) | 15.0 (13.0-19.0 | 16.0 (13.0-21.0) | 17.0 (14.0-21.0) | <0.001 |
| Total cholesterol (mmol/L) | 4.5 (4.0-5.0) | 4.5 (4.0-5.0) | 4.5 (4.1-5.1) | 0.127 |
| Triglycerides (mmol/L) | 1.3 (0.9-1.8) | 1.4 (1.0-2.0) | 1.5 (1.1-2.1) | <0.001 |
| LDL (mmol/L) | 2.5 (2.1-2.8) | 2.5 (2.1-2.9) | 2.5 (2.2-3.0) | 0.053 |
| HDL (mmol/L) | 1.2 (1.0-1.4) | 1.1 (1.0-1.3) | 1.1 (1.0-1.3) | <0.001 |
| SUA(μmol /L) | 256.9 (212.3-312.6) | 281.7 (228.3-333.9) | 305.0 (259.7-355.4) | <0.001 |
| Serum creatinine (μmol /L) | 72.7 (64.3-82.9) | 76.4 (67.1-86.4) | 81.2 (72.1-88.1) | <0.001 |
| Urine creatinine (μmol/L) | 7388.0 (4146.5-12449.0) | 7857.0 (4680.5-12943.5) | 8552.0 (4629.0-13894.5) | 0.052 |
| mALB (mg/L) | 6.3 (3.5-11.7) | 8.3 (4.6-14.5) | 9.6 (5.0-19.5) | <0.001 |
| eGFR (mL/min/1.73m^2^) | 97.7 (87.0-110.2) | 96.2 (85.9-109.9) | 95.6 (86.7-108.3) | 0.474 |
| uACR (mg/g) | 7.7 (5.3-12.5) | 8.8 (5.8-15.3) | 10.0 (6.3-19.7) | <0.001 |

Continuous variables were shown as mean±SD if normally distributed or median (quartile 1, quartile 3) if non-normally distributed. Categorical variables were expressed as numbers and percentages of subjects. The Mann–Whitney test was used for non-normally distributed continuous variables. Differences between groups of categorical variables were compared with chi-squared tests. BMI, body mass index; SBP, systolic blood pressure; DBP, diastolic blood pressure; GLU, fasting plasma blood glucose; ALT, alanine transaminase; AST, aspartate-aminotransferase; LDL, low-density lipoprotein; HDL, high-density lipoprotein; SUA, serum uric acid; eGFR, estimated glomerular filtration rate; mALB , Urine albumin; uACR, uACR, urinary albumin-to-creatinine ratio
