# Supplementary material for: Sex differences in impact of cumulative systolic blood pressure from childhood to adulthood on albuminuria in midlife: a 30-year prospective cohort study
Source: BMC Public Health. 2023 Apr 11;23:666. doi: 10.1186/s12889-023-15613-y (PMC10088136; doi:10.1186/s12889-023-15613-y)
Supplement: Supplementary file 4 — Supplementary Material 4 [file 12889_2023_15613_MOESM4_ESM.docx]

| **Additional file 4.** Demographic and clinical characteristics of the participants at baseline and the latest follow-up, by incremental AUC of SBP | | | | |
| --- | --- | --- | --- | --- |
| **Characteristics** | **Low**  **(AUC< 7.98)** | **Middle**  **(7.98≤AUC≤19.90)** | **High**  **(AUC >19.90)** | ***P* value** |
| Males (n, %) | 206 (36.7%) | 329 (58.6%) | 443 (79.0%) | <0.001 |
| n (%) of albuminuria | 50 (8.9%) | 64 (11.4%) | 76 (13.5%) | 0.049 |
| **Childhood** |  |  |  |  |
| Age (years) | 13.0 (10.0-15.0) | 12.0 (9.0-14.0) | 12.0 (9.0-14.0) | <0.001 |
| Height (cm) | 146.2 (129.0-154.6) | 137.0 (123.9-151.2) | 135.5 (124.0-147.6) | <0.001 |
| Weight (kg) | 37.5 (25.2-45.6) | 29.5 (22.5-40.6) | 29.2 (23.0-37.25) | <0.001 |
| BMI (kg/m^2^) | 17.2 (15.1-19.0) | 15.9 (14.7-17.8) | 15.9 (14.8-17.3) | <0.001 |
| SBP (mmHg) | 109.3 (103.0-117.3) | 102.7 (96.7-110.7) | 100.0 (92.7-106.45) | <0.001 |
| DBP (mmHg) | 68.6 (61.3-74.0） | 64.0 (60.0-70.7) | 62.0 (57.3-70.0) | <0.001 |
| **Adulthood** |  |  |  |  |
| Age (years) | 45.0 (41.0-46.0) | 43.0 (40.0-46.0) | 43.0 (41.0-45.0) | <0.001 |
| Current smoking (n, %) | 160 (28.5%) | 258 (46.0%) | 350 (62.4%) | <0.001 |
| Alcohol consumption (n, %) | 108 (19.3%) | 191 (34.0%) | 210 (37.4%) | <0.001 |
| Exercise (n, %) | 189 (33.7%) | 144 (25.7%) | 132 (23.5%) | <0.001 |
| Hypertension (n, %) | 31 (5.5%) | 62 (11.1%) | 110 (19.6%) | <0.001 |
| Diabetes mellitus (n, %) | 16 (2.9%) | 15 (2.7%) | 24 (4.3%) | 0.254 |
| Hyperlipidaemia (n, %) | 53 (9.4%) | 45 (8.0%) | 74 (13.2%) | 0.013 |
| BMI (kg/m^2^) | 23.2 (21.5-25.4) | 23.9 (21.9-26.0) | 24.5 (22.3-26.8) | <0.001 |
| Waist (cm) | 82.5 (76.5-89.2) | 84.5 (78.5-91.4) | 87.9 (80.2-94.1) | <0.001 |
| Hips (cm) | 91.4 (88.2-94.8) | 92.2 (89.0-95.6) | 92.8 (89.4-96.6) | <0.001 |
| SBP (mmHg) | 117.7 (109.3-126.3) | 121.3 (112.7-132.0) | 126.3 (117.4-137.3) | <0.001 |
| DBP (mmHg) | 73.7 (67.3-80.3) | 76.0 (68.7-84.7) | 80.2 (73.0-87.9) | <0.001 |
| Fasting glucose (mmol/L) | 4.6 (4.3-4.9) | 4.6 (4.3-4.9) | 4.6 (4.3-5.0) | 0.086 |
| ALT (U/L) | 17.0 (13.0-24.0) | 19.0 (14.0-27.5) | 22.0 (16.0-32.0) | <0.001 |
| AST (U/L) | 15.0 (13.0-20.0) | 16.0 (13.0-20.0) | 17.0 (14.0-22.0) | <0.001 |
| Total cholesterol (mmol/L) | 4.5 (4.1-5.0) | 4.4 (4.0-4.9) | 4.6 (4.1-5.2) | 0.025 |
| Triglycerides (mmol/L) | 1.3 (1.0-1.9) | 1.4 (1.0-1.9) | 1.5 (1.1-2.1) | <0.001 |
| LDL (mmol/L) | 2.5 (2.1-2.9) | 2.5 (2.1-2.9) | 2.6 (2.2-3.0) | 0.004 |
| HDL (mmol/L) | 1.2 (1.0-1.4) | 1.1 (1.0-1.3) | 1.1 (1.0-1.3) | <0.001 |
| SUA (μmol /L) | 225.0 (211.6-305.8) | 285.3 (229.2-343.9) | 303.9 (258.9-357.9) | <0.001 |
| Serum creatinine (μmol/L) | 72.3 (64.3-82.8) | 77.0 (68.2-86.8) | 80.1 (71.1-88.7) | <0.001 |
| Urine creatinine (μmol/L) | 7328.0 (4208.0-12241.0) | 7755.0 (4469.0-12667.5) | 8654.0 (4755.5-14003.0) | 0.014 |
| mALB (mg/L) | 6.8 (3.7-12.7) | 8.0 (4.5-13.7) | 9.3 (4.7-17.4) | <0.001 |
| eGFR (mL/min/1.73m^2^) | 96.5 (86.8-109.0) | 96.4 (86.2-109.6) | 95.9 (86.4-109.6) | 0.955 |
| uACR (mg/g) | 8.3 (5.5-14.0) | 8.8 (5.8-15.4) | 9.1 (5.8-17.6) | 0.020 |

Continuous variables were shown as mean±SD if normally distributed or median (quartile 1, quartile 3) if non-normally distributed. Categorical variables were expressed as numbers and percentages of subjects. The Mann–Whitney test was used for non-normally distributed continuous variables. Differences between groups of categorical variables were compared with chi-squared tests. BMI, body mass index; SBP, systolic blood pressure; DBP, diastolic blood pressure; GLU, fasting plasma blood glucose; ALT, alanine transaminase; AST, aspartate-aminotransferase; LDL, low-density lipoprotein; HDL, high-density lipoprotein; SUA, serum uric acid; eGFR, estimated glomerular filtration rate; mALB , Urine albumin; uACR, uACR, urinary albumin-to-creatinine ratio
