# Supplementary material for: Sex differences in impact of cumulative systolic blood pressure from childhood to adulthood on albuminuria in midlife: a 30-year prospective cohort study
Source: BMC Public Health. 2023 Apr 11;23:666. doi: 10.1186/s12889-023-15613-y (PMC10088136; doi:10.1186/s12889-023-15613-y)
Supplement: Supplementary file 5 — Supplementary Material 5 [file 12889_2023_15613_MOESM5_ESM.docx]

**Additional file 5.** Association of total AUC groups of DBP with midlife albuminuria by sex

| **Characteristics** | **N (%)** | **Unadjusted** | | **Model 1** | | **Model 2** | |  |
| --- | --- | --- | --- | --- | --- | --- | --- | --- |
|  |  | **OR(95%CI)** | ***P* value** | **OR(95%CI)** | ***P* value** | **OR(95%CI)** | ***P* value** | ***P**** |
| **Total subjects** |  |  |  |  |  |  |  |  |
| Low | 44 (7.8%) | 1 | - | 1 | - | 1 | - | <0.001 |
| Middle | 52 (9.3%) | 1.20 (0.79-1.83) | 0.394 | 1.18 (0.76-1.84) | 0.467 | 1.21 (0.77-1.92) | 0.413 |  |
| High | 94 (16.8%) | 2.37 (1.62-3.46) | <0.001^#^ | 1.89 (1.21-2.97) | 0.005^#^ | 1.85 (1.16-2.95) | 0.009^#^ |  |
| **Male Subjects** |  |  |  |  |  |  |  |  |
| Low | 17 (7.0%) | 1 | - | 1 | - | 1 | - |  |
| Middle | 18 (5.6%) | 0.78 (0.39-1.54) | 0.474 | 0.74 (0.36-1.52) | 0.407 | 0.89 (0.41-1.92) | 0.760 |  |
| High | 66 (16.0%) | 2.53 (1.44-4.42) | 0.001^#^ | 1.92 (1.02-3.62) | 0.045 | 2.12 (1.07-4.23) | 0.051 |  |
| **Female Subjects** |  |  |  |  |  |  |  |  |
| Low | 27 (8.5%) | 1 | - | 1 | - | 1 | - |  |
| Middle | 34 (14.3%) | 1.81 (1.06-3.10) | 0.030 | 1.74 (0.98-3.09) | 0.058 | 1.69 (0.95-3.03) | 0.076 |  |
| High | 28 (18.8%) | 2.50 (1.42-4.42) | 0.002^#^ | 1.59 (0.80-3.14) | 0.187 | 1.49 (0.74-3.01) | 0.268 |  |

Model 1: adjusted for age, gender, systolic blood pressure, body mass index at baseline, body mass index, waist, hips, smoking, alcohol consumption, exercise, and history of hypertension, diabetes, hyperlipidaemia in 2017.

Model 2: adjusted for model 1+ fasting blood glucose, ALT, AST, total cholesterol, triglycerides, low-density lipoprotein, high-density lipoprotein, serum uric acid and serum creatinine.

*P**: *p* value for interaction terms of incremental AUC and sex

^#^: *p* value was considered significant after false discovery rate correction
