# Supplementary material for: Sex differences in impact of cumulative systolic blood pressure from childhood to adulthood on albuminuria in midlife: a 30-year prospective cohort study
Source: BMC Public Health. 2023 Apr 11;23:666. doi: 10.1186/s12889-023-15613-y (PMC10088136; doi:10.1186/s12889-023-15613-y)
Supplement: Supplementary file 7 — Supplementary Material 7 [file 12889_2023_15613_MOESM7_ESM.docx]

| **Additional file 7.** Association of smoking status with midlife albuminuria, by sex | | | | |
| --- | --- | --- | --- | --- |
| **Smoking** | **N (%)** | **OR values** | **95% CI** | ***P* value** |
| **Total subjects** |  |  |  |  |
| no | 915 (54.4%) | 1 | - | - |
| yes | 768 (45.6%) | 0.92 | 0.68 - 1.24 | 0.567 |
| **Male Subjects** |  |  |  |  |
| no | 236 (24.1%) | 1 | - | - |
| yes | 742 (75.9%) | 1.42 | 0.84 - 2.39 | 0.189 |
| **Female Subjects** | |  |  |  |
| no | 616 (87.4%) | 1 | - | - |
| yes | 89 (12.6%) | 0.27 | 0.04 - 2.01 | 0.200 |
